# Representation learning in intraoperative vital signs for heart failure risk prediction

**DOI:** 10.1186/s12911-019-0978-6

**Published:** 2019-12-09

**Authors:** Yuwen Chen, Baolian Qi

**Affiliations:** 1 Chengdu Institute of Computer Applications, Chinese Academy of Sciences, Chengdu, China; 2 Chongqing Institute of Green and Intelligent Technology, Chinese Academy of Sciences, Chongqing, China; 3 University of Chinese Academy of Sciences, Beijing, China

**Keywords:** Heart failure, Perioperative period, Machine learning

## Abstract

**Background:**

The probability of heart failure during the perioperative period is 2% on average and it is as high as 17% when accompanied by cardiovascular diseases in China. It has been the most significant cause of postoperative death of patients. However, the patient is managed by the flow of information during the operation, but a lot of clinical information can make it difficult for medical staff to identify the information relevant to patient care. There are major practical and technical barriers to understand perioperative complications.

**Methods:**

In this work, we present three machine learning methods to estimate risks of heart failure, which extract intraoperative vital signs monitoring data into different modal representations (statistical learning representation, text learning representation, image learning representation). Firstly, we extracted features of vital signs monitoring data of surgical patients by statistical analysis. Secondly, the vital signs data is converted into text information by Piecewise Approximate Aggregation (PAA) and Symbolic Aggregate Approximation (SAX), then Latent Dirichlet Allocation (LDA) model is used to extract text topics of patients for heart failure prediction. Thirdly, the vital sign monitoring time series data of the surgical patient is converted into a grid image by using the grid representation, and then the convolutional neural network is directly used to identify the grid image for heart failure prediction. We evaluated the proposed methods in the monitoring data of real patients during the perioperative period.

**Results:**

In this paper, the results of our experiment demonstrate the Gradient Boosting Decision Tree (GBDT) classifier achieves the best results in the prediction of heart failure by statistical feature representation. The sensitivity, specificity and the area under the curve (AUC) of the best method can reach 83, 85 and 84% respectively.

**Conclusions:**

The experimental results demonstrate that representation learning model of vital signs monitoring data of intraoperative patients can effectively capture the physiological characteristics of postoperative heart failure.

## Background

Heart failure occurs when the heart is unable to pump sufficiently to maintain blood flow to meet the body’s needs. Signs and symptoms commonly include shortness of breath, excessive tiredness and leg swelling. It has been considered as one of the deadliest human diseases worldwide, and the accurate prediction of this risk would be vital for heart failure prevention and treatment. It is estimated in the “Report on Cardiovascular Disease in China, 2018” by China Cardiovascular Center that more than 290 million people suffer from heart failure. Cardiovascular disease has become the leading cause of death for residents, accounting for more than 40% of total. Data from China Health Yearbook 2018 indicated that there are over 50 million operations each year in China, in which the perioperative adverse cardiac events have reached 2%. The incidence of adverse events in heart failure patients during surgery is 2–17%, which has become the most important reason for perioperative complications and mortalities, significantly higher than other patients (0.1–0.2%). At present, there is a lack of early intraoperative prediction techniques for perioperative adverse cardiac events. In addition to the basic Electrocardiograph (ECG), ST segment, ABP monitoring methods, researchers also utilized experimental indicators such as BMP9, neutrophil-lymphocyte ratio, creatine kinase isoenzyme stratification, having a certain evaluation effect on postoperative adverse cardiac events. However, it is difficult to predict early diagnosis and prediction because of obvious hysteresis, so it is often used in the postoperative diagnosis of adverse events. Therefore, the early clinical diagnosis of adverse events of heart failure still relies on the clinical experience of anesthesiologists and physicians.

Currently, the research on heart failure is mainly based on the data from patients’ medical records, physical characteristics, auxiliary examination, the treatment plan, and the algorithm is used to build the model for studying, analyzing and classifying of diagnosis and prediction. In addition, most studies mainly analyzed the characteristics of electrocardiogram data and built the diagnostic model of heart failure [1–6]. Choi et al. [7] used the recurrent neural network algorithm to analyze the diagnostic data of patients with heart failure, including time series of doctor’s orders, spatial density and other characteristics, to build a diagnostic model of heart failure, and verified by experiment that the area under the curve (AUC) of the diagnosis of this model was 0.883. Koulaouzidis [8] used Naive Bayes algorithm to analyze the patients with heart failure in the last hospitalization and remote monitoring data, including patient’s condition, cause of heart failure, complications, the examination, the New York Heart Association (NYHA) Functional Classification, treatment, and remote monitoring data (e.g., vital signs, body weight, treatment, alcohol consumption and general situation), and built the prediction model of the readmission of patients with heart failure, the predicted AUC reached 0.82 after followed-up of (286 + 281) d. Shameer et al. [9] also utilized Naive Bayes algorithm to analyze about data variables of patients with heart failure, including diagnosis data, treatment data, examination data, records of doctor’s orders, and vital signs data, and built a model for predicting readmission of patients with heart failure, with a predicted AUC of 0.78. Zheng et al. [10] presented a method used support vector machine algorithm to analyze the data of patients with heart failure, including age, type of medical insurance, sensitivity assessment (audio-visual and thinking), complications, emergency treatment, the drug-induced risks, the period of last hospitalization, and built a prediction model for the readmission of patients with heart failure, with a prediction accuracy of 78.4%. Chen et al. [11] analyzed 24 h dynamic electrocardiogram of heart failure patients and healthy controls by using support vector machine (SVM) algorithm based on non-equilibrium decision tree. The paper first cut electrocardiogram into segments of more than 5 min, then analyzed the heart rate variability with RR interval series and built a model of heart failure severity classification, which achieved the classification accuracy of 96.61%.

As far as we know that there is no research on the prediction of perioperative heart failure risk of patients by directly using intraoperative vital signs monitoring data. However, previous studies have shown that the intraoperative direct monitoring data has the significant value of early diagnosis and early warning after preprocessing and analyzing the time series data. Matthew et al. [12] presented that 30% of critical cardiovascular events have abnormal monitoring signs in 24 h before the cardiovascular critical event. In another study, the paper [13] analyzed 5 vital signs data of patients, and the deterioration of its indicators could warn the doctor of respiratory failure. Petersen provided a model to predict further treatment in the ICU of the patient with monitoring data, and its early warning sensitivity was 0.42 [14]. Therefore, we used intraoperative vital signs monitoring data to predict the risk of perioperative heart failure. However, the clinical information is far beyond the processing capacity of human brains because of its high rate of production and large amount, and the rapid change of the patient’s condition. A lot of clinical information can make it difficult for medical staff to identify the information relevant to patient care. Since machine learning is a kind of algorithm that automatically analyzes and obtains rules from data and uses rules to predict unknown data, we used machine learning to build the model for heart failure risk prediction. Thus, in this paper, we mainly used five indicators, including the intraoperative monitoring heart rate, diastolic blood pressure, systolic blood pressure, blood oxygen saturation, pulse pressure difference to learn statistical feature representation, text feature representation and image feature representation of vital sign monitoring data, and then these features were then input into the classifier to predict perioperative heart failure.

Our main contributions are in two areas: 1) To our knowledge, ours is the first study to predict perioperative heart failure using only intraoperative vital signs monitoring data, unlike other studies that used ECG data and bio-marker as input to a classifier. 2) Our methods create meaningful representations of vital signs monitoring data, we present three examples of representation learning, with a focus on representations that work for heart failure prediction.

The rest of this paper is organized as follows: The preliminary and related technology, and methodology of this paper is discussed in Section 2. The Section 3 reports the experimental results, and the Section 4 discusses the implications and highlights limitations of the study. Finally, the Section 5 discusses the conclusion of this paper.

## Methods

In order to provide a common understanding throughout the text, this section describes the concept of PAA, SAX, LDA, GRTS and CNN algorithms utilized as feature extraction techniques and time series classification algorithms, which is implemented in the proposed approach.

### Time series classification (TSC)

Classification of unlabeled time series into existing classes is a traditional data mining task. All classification methods start by establishing a classification model based on labeled time series. In this case, “labeled time series” means that we build the model using a training dataset with the correct classification of observations or time series. The model is then used to predict a new, unlabeled observations or time series. Prediction of heart failure risk is summarized as a multidimensional time series classification problem. TSC is an important and challenging problem in data mining. With the increase of time series data availability, hundreds of TSC algorithms have been proposed [15, 16]. The time series classification problem is generally composed of extracting time series feature representation and machine learning classification algorithm. The methods used in this paper are the decision tree algorithm [17, 18], gradient boosting machine algorithm [19, 20], logistic regression algorithm [21], Bayesian algorithm [22], SVM [23], random forest [24] and popular deep learning methods [25, 26].

### Piecewise approximate aggregation (PAA)

**Fig. 1:**
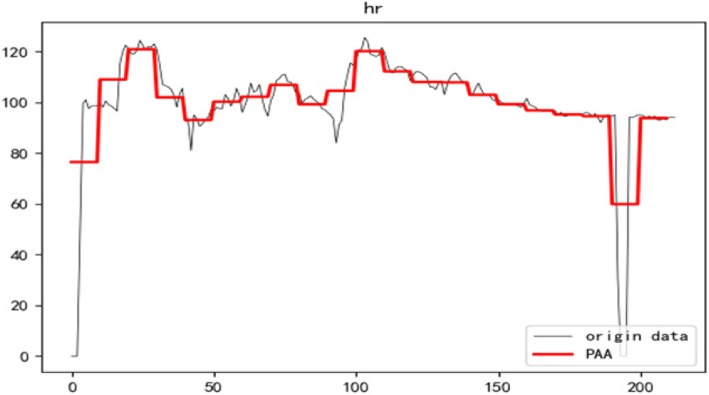
The PAA representation of time series data

Piecewise Approximate Aggregation was originally a time series data representation method proposed by Lin et al. [27]. It can significantly reduce the dimensionality of the data while maintaining the lower bound of distance measurement in Euclidean space. Assume that the original time series is **C** = {***x***_**1**,_***x***_**2**_, …***x***_***N***_}, the sequence defines that the PAA is $\overline{\boldsymbol{C}}=\left\{{\overline{\boldsymbol{x}}}_{\mathbf{1}},{\overline{\boldsymbol{x}}}_{\mathbf{2}}\dots .{\overline{\boldsymbol{x}}}_{\boldsymbol{w}}\right\}$. Figure 1 shows the PAA of patient heart rate time series in this article. The Formula as Eq. 1.

$$ {\overline{x}}_i=\frac{\omega }{N}\bullet \sum \limits_{j=\frac{N}{\omega}\left(i-1\right)+1}^{\frac{N}{\omega }i}{x}_j\ (1) $$

### Symbolic aggregate approximation (SAX)

**Fig. 2:**
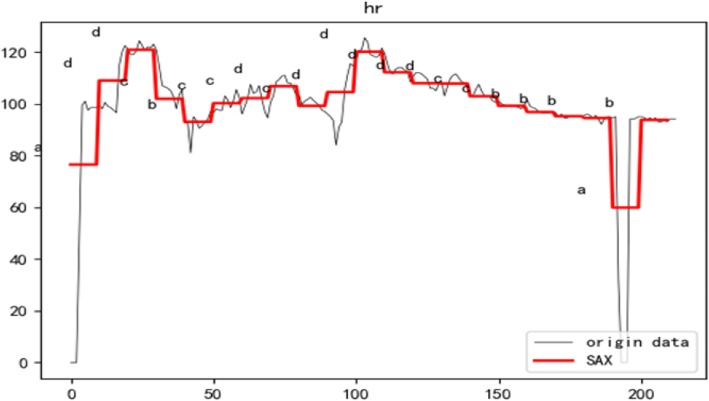
The SAX representation of time series data

Symbolic Aggregate Approximation [27] was a time-series data representation method that Lin et al. extended the PAA-based method to obtain the symbol and time series features in the discretized symbol representation of the PAA feature representation of a time series. Figure 2 shows the sax representation of the patient’s heart rate. The red line shows the data that has been aggregated with the PAA. For each coefficient, we assign the literal associated with the area.

### Latent Dirichlet allocation (LDA)

**Fig. 3:**
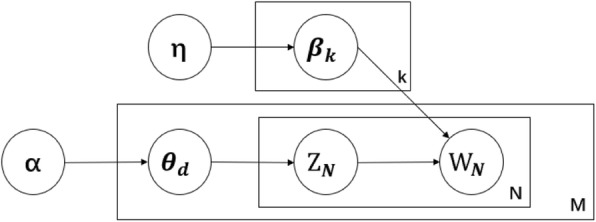
The plate model representation of LDA

Latent Dirichlet Allocation [28] was proposed by Blei David in 2003 to estimate the subject distribution of the document. It gives a probability distribution to the topics of each document in the document set, so that by analyzing some documents to extract their topic distribution, you can cluster topics or classify text based on the topic distribution. See Formula 2 and Fig. 3. Here k is the number of topics (fixed on initialization of the LDA model), M is the number of documents, N is the number of words in the document, which itself is represented by the vector w as a bag-of-words. The ***β***_***k***_ is the multinomial distribution words that represent the topics and is drawn from the prior Dirichlet distribution with the parameter η. Similarly, the topic distribution ***θ***_***d***_ is drawn from a Dirichlet prior with the parameter α. The ***z***_***ij***_ is the topic which is most likely to have generated ***w***_***ij***_, which is the j-th word in the i-th document. In this paper, the topic model is used to extract the text features of patient’s sign monitoring data. Specifically, the time series of vital signs is converted into symbols by SAX, these symbols are then transformed into human-readable text using high-level semantic abstraction. Finally, LDA model is used to extract text topics of patients for heart failure prediction. See below for details in section 3.

2 $$ p\left(\theta, \boldsymbol{z}|\boldsymbol{w},\alpha, \beta \right)=\frac{p\left(\theta, \boldsymbol{z},\boldsymbol{w}|\alpha, \beta \right)}{p\left(\boldsymbol{w}|\alpha, \beta \right)} $$

### Grid representation for time series (GRTS)

**Fig. 4:**
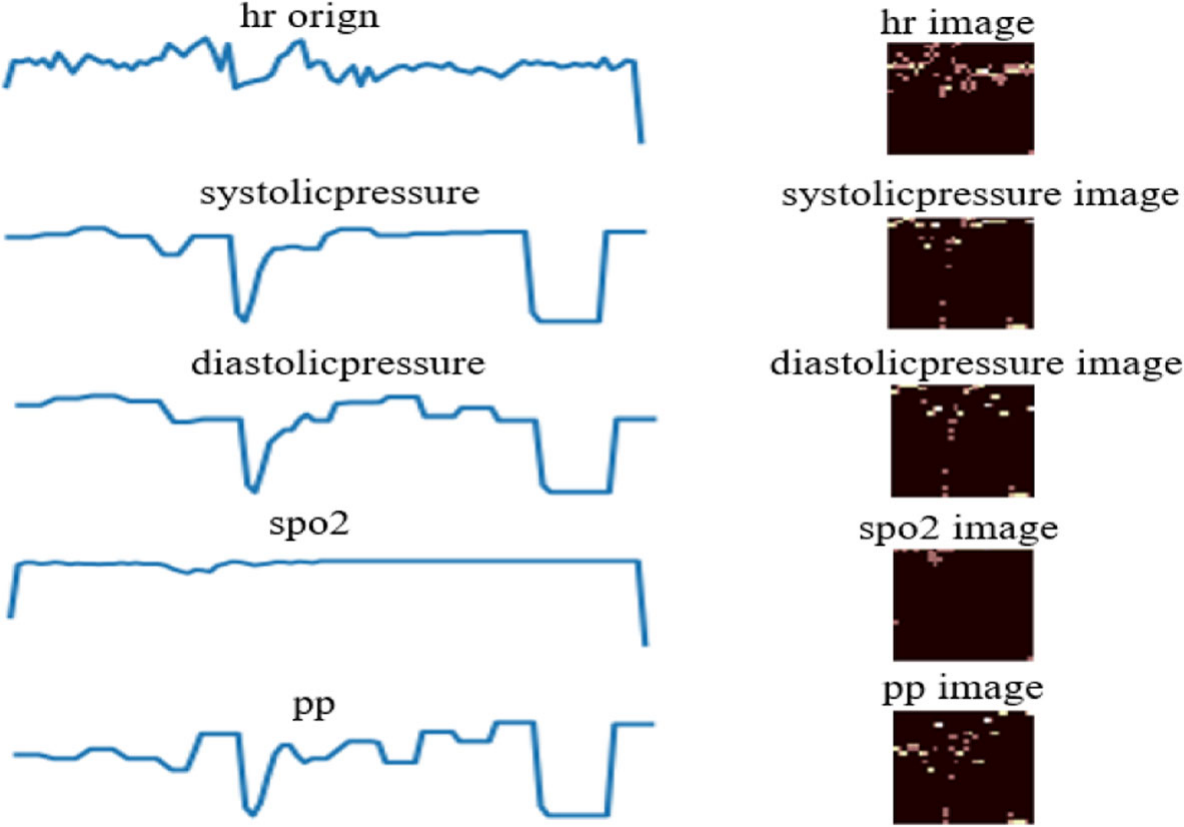
Grid representation for time series

The time series grid representation is an algorithm for converting time series data into images, which introduces a m × n grid structure to partition time series. According to the characteristics of time and value, the points in time series are assigned to their corresponding rectangles. The grid is then compiled into a matrix where each element is the number of points in the corresponding rectangle. The matrix form not only can reflect the point distribution characteristic of the sequence, but also improve the computational efficiency by using the sparse matrix operation method. See the algorithm for details [29]. Figure 4 demonstrates the schematic diagram of converting patient’s heart rate, diastolic blood pressure, systolic pressure, and pulse pressure difference time series data into a grid representation.

### Convolutional neural network (CNN)

**Fig. 5:**
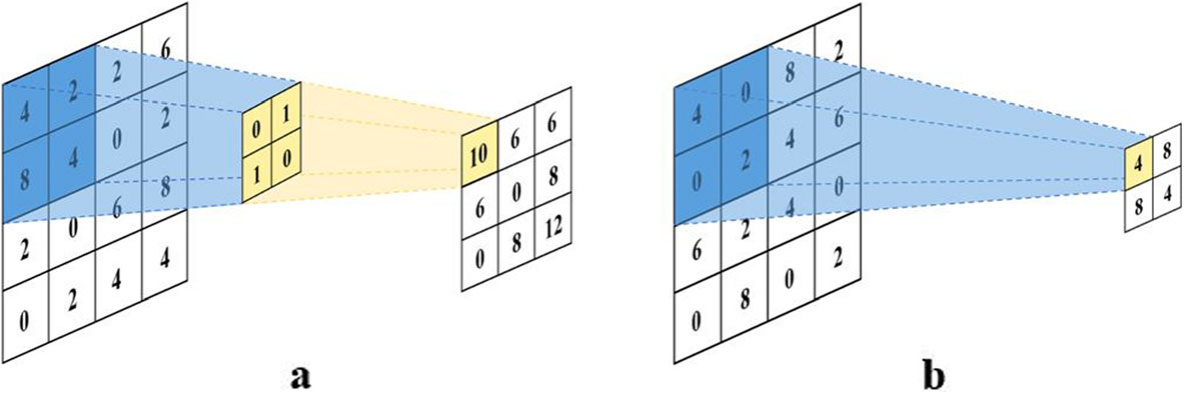
**a** The convolution operation of Convolutional Neural Networks. **b** The pooling operation of Convolutional Neural Networks

In recent year, deep learning (DL) models have achieved a high recognition rate for computer vision [30, 31] and speech recognition [32]. A Convolutional Neural Network is one of the most popular DL models. Unlike the traditional feature-based classification framework, CNN does not require hand-crafted features. Both feature learning and classification parts are integrated in a model and are learned together. Therefore, their performances are mutually enhanced. Related CNN algorithms can be found in [33]. The two most essential components of CNN are the convolution (Conv) layer and pooling (Pool) layer. Figure 5: a shows that the convolution layer realizes the convolution operation, and extracts the image features by calculating the inner product of the input image matrix and the kernel matrix. The other essential component is the pooling layer, also known as the sub-sampling layer, which is primarily responsible for simpler tasks. Figure 5: b shows that the pooling layer only retains part of the data after the convolution layer. It reduces the number of significant features extracted by the convolution layer and refines the retained features. In this paper, CNN is used to extract the image features of the vital signs monitoring data from surgical patients.

### Representation learning for heart failure risk prediction

**Fig. 6:**
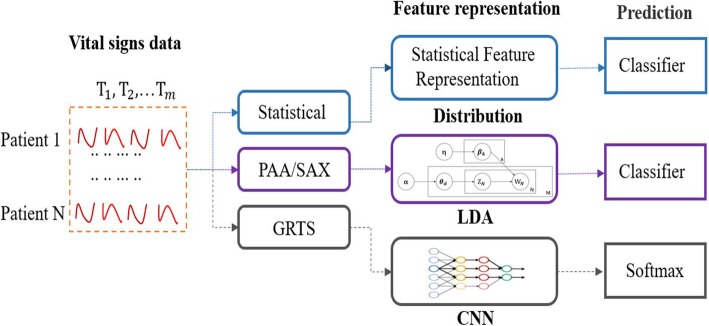
The overall workflow of the proposed method

This section mainly demonstrates how to use the different time series feature representation of vital signs during surgery to predict the risk of postoperative heart failure using the relevant techniques described above. First a general overview over the workflow is given and shown in Fig. 6. Then each of the components are described in more detail in individual subsections.

The overall workflow of our presented method consists of three representation techniques towards heart failure which are described in more detail in the following Sections. They are:

Statistical representation of vital signs data: Statistical analysis of vital signs monitoring data of surgical patients to extract features for heart failure prediction.

Text representation of vital signs data: Firstly, the time series of vital signs is converted into symbols by the SAX, these symbols are then transformed into human-readable text using high-level semantic abstraction. Finally, the LDA model is used to extract text topics of patients for heart failure prediction.

Image representation of vital signs data: The vital sign monitoring time series data of the surgical patient is converted into a grid image by using the grid representation, and then the convolutional neural network is directly used to identify the grid image for heart failure prediction.

**Fig. 7:**
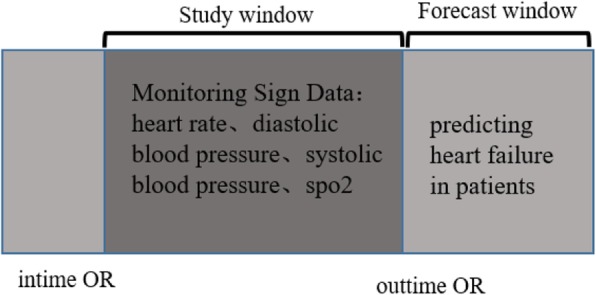
Learning and prediction diagram

Perioperative heart failure prediction is based only on vital signs monitoring data of intraoperative patients. Indicators include heart rate (HR/hr), systolic blood pressure (NISYSBP/nisysbp), diastolic blood pressure (NIDIASBP/nidiasbpe), SpO2 (spo2), and pulse pressure difference (PP/pp). Learning window: defined as the duration of continuous monitoring during surgery, predictive window: defined as the patient’s perioperative period. As shown in Fig. 7.

### Statistical representation of vital signs data

In order to capture the various statistical feature of patient monitoring data trends, and mine intraoperative patient monitoring data from multiple dimensions in this paper, the mean (mean), variance (std), minimum (min), maximum (max), 25% (perc25), 50% (perc50), 75% (perc75) quantile, skewness (skew), kurtosis (kurt) and derivative variables of the first order difference (diff) of each monitoring index were calculated. That is, a total of 90 statistical parameters are obtained as derivative variables. The individual characteristic derivative variables are shown in Table 1, and the calculation is shown in Eq. 3. Finally, the classifier is used to predict heart failure. Specifically, the meaning of Feature variables in Table 1 are connected the abbreviation use “_” to add abbreviation together. For example: “mean_hr” means the mean of heart rate (hr), “min_diff_hr” means the minimum of the first order difference of heart rate, and “perc25_nisysbp” means that 25% of systolic blood pressure.

**Table 1:** Overview about non-invasive physiological parameters and related feature variables

NIPP	Feature variables
HR	mean_hr, std_hr, min_hr, perc25_hr, perc50_hr, perc75_hr, max_hr, mean_diff_hr, std_diff_hr, min_diff_hr, perc25_diff_hr, perc50_diff_hr, perc75_diff_hr, max_diff_hr, skew_hr, kurt_hr, diff-skew_diff_hr, diff-kurt_diff_hr
NISYSBP	mean_nisysbp, std_nisysbp, min_nisysbp, perc25_nisysbp, perc50_nisysbp, perc75_nisysbp, max_nisysbp, mean_diff_nisysbp, std_diff_nisysbp, min_diff_nisysbp, perc25_diff_nisysbp, perc50_diff_nisysbp, perc75_diff_nisysbp, max_diff_nisysbp, skew_nisysbp, kurt_nisysbp, diff-skew_diff_nisysbp, diff-kurt_diff_nisysbp
NIDIASBP	mean_nidiasbpe, std_nidiasbpe,min_nidiasbpe, perc25_nidiasbpe, perc50_nidiasbpe, perc75_nidiasbpe, max_nidiasbpe, mean_diff_nidiasbpe, std_diff_nidiasbpe, min_diff_nidiasbpe, perc25_diff_nidiasbpe, perc50_diff_nidiasbpe, perc75_diff_nidiasbpe, max_diff_nidiasbpe, skew_nidiasbpe, kurt_nidiasbpe, diff-skew_diff_nidiasbpe, diff-kurt_diff_nidiasbpe
SPO2	mean_spo2, std_spo2,min_spo2, perc25_spo2, perc50_spo2, perc75_spo2, max_spo2, mean_diff_spo2, std_diff_spo2, min_diff_spo2, perc25_diff_spo2, perc50_diff_spo2, perc75_diff_spo2, max_diff_spo2, skew_spo2, kurt_spo2, diff-skew_diff_spo2, diff-kurt_diff_spo2
PP	mean_pp, std_pp, min_pp, perc25_pp, perc50_pp, perc75_pp, max_pp, mean_diff_pp, std_diff_pp, min_diff_pp, perc25_diff_pp, perc50_diff_pp, perc75_diff_pp, max_diff_pp, skew_pp, kurt_pp, diff-skew_diff_pp

$$ \mu =\frac{1}{T}\sum \limits_{i=1}^T{x}_i $$

$$ {\sigma}^2=\sum \limits_{i=1}^T\frac{1}{T}{\left({x}_i-\mu \right)}^2 $$

$$ \mathrm{skewness}\left(\mathrm{X}\right)=E\left[{\left(\frac{X-\mu }{\sigma}\right)}^3\right]=\frac{1}{T}\sum \limits_{i=1}^T\frac{{\left({x}_i-\mu \right)}^3}{\sigma^3} $$

3 $$ \mathrm{kurtosis}\left(\mathrm{X}\right)=E\left[{\left(\frac{X-\mu }{\sigma}\right)}^4\right]=\frac{1}{T}{\sum}_{i=1}^T\frac{{\left({x}_i-\mu \right)}^4}{\sigma^4} $$

$$ {Q}_{25\%}=\frac{n+1}{4} $$

$$ {Q}_{50\%}=\frac{2\left(n+1\right)}{4}=\frac{n+1}{2} $$

$$ {Q}_{75\%}=\frac{3\left(n+1\right)}{4} $$

### Text representation of vital signs data

**Fig. 8:**
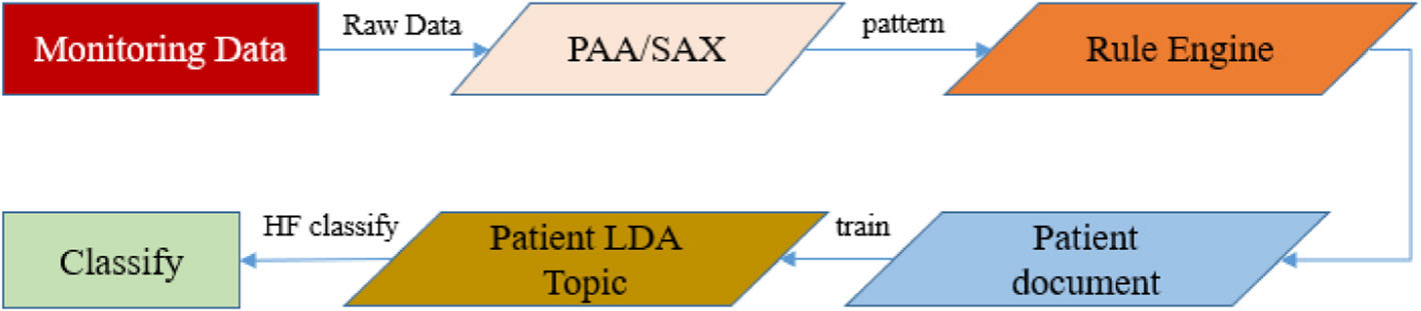
Prediction of heart failure risk based on text features

The second method in this paper is based on the textual features of patient monitoring data for heart failure prediction. The specific process is shown in Fig. 8. These include the following steps:

1. Normalization: Normalize the sign data to the mean 0 and variance 1.
2. Segmentation: Use the PAA to segmentation patient vital sign data.
3. Alphabetization of Symbols: Use the SAX to Symbolize patient vital sign data.
4. Textualization: Use the rules engine to textual Symbolic alphabetized data.
5. Topic clustering: Use the LDA to cluster all patient text data topics.
6. Prediction: Predicting heart failure based on probability distribution of each patient’s topic.

The advantage of textualization is that the results of the analysis are easier for humans to understand. Though the alphabetization of Symbols obtained from the SAX pattern extraction give a representation of the shape of the data within the time frame, the SAX strings are not intuitively understood and still have to be interpreted. Furthermore, by considering the statistics of the time frame in the abstract process, we are able to represent more information in the text than just the shape. Therefore, we use a rule-based engine that uses the SAX patterns and the statistical information of the time frame to produce text that is understandable to humans. The general form of the rules is given in Eq. 4 where < pattern > is the SAX pattern, < l > is the level, < f > is the feature, < mod > is a modifier for the pattern movement and < pm > is the pattern movement. Eq. 5 shows the possible values that the individual output variables can take.

4 $$ \left\{<\mathrm{pattern}>\right\}=\left\{<\mathrm{l}><\mathrm{f}><\operatorname{mod}><\mathrm{pm}>\right\} $$

<l > = [‘low’,‘medium’,‘high’].

<f > = The values are shown in Table 1.

5 $$ <\operatorname{mod}>=\left[`\mathrm{slowly}',`\mathrm{rapidly}',`\mathrm{upward}',`\mathrm{downward}'\right] $$

<pm> = [‘decreasing’,‘increasing’,‘steady’,‘peak’,‘varying’].

**Fig. 9:**
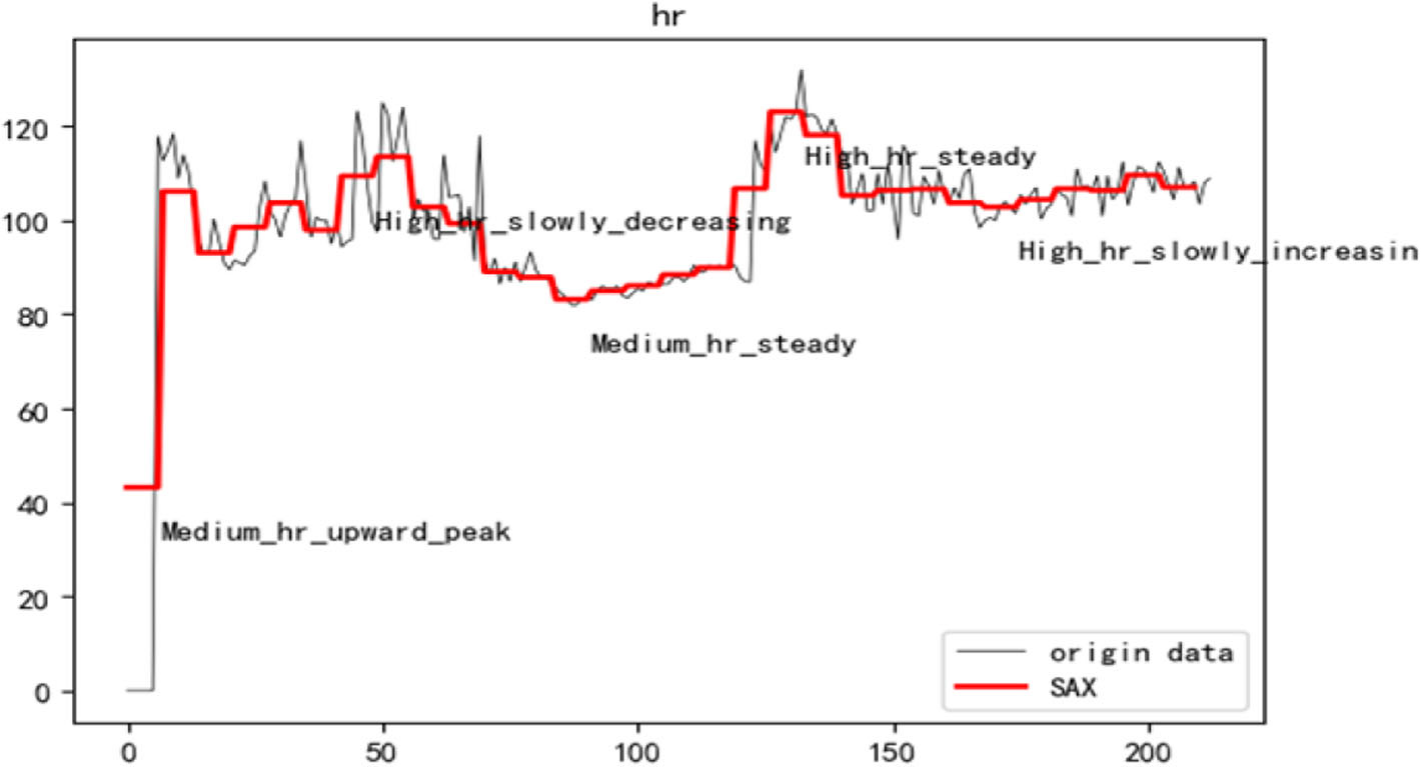
The text representation of vital signs data

The heart rate, diastolic blood pressure, systolic blood pressure, spo2 and pulse pressure difference of the surgical patients are converted into text semantics. See Fig. 9. The patient text topic is extracted through the LDA, and finally the risk of heart failure is predicted by the classifier.

### Image representation of vital signs data

**Fig. 10:**
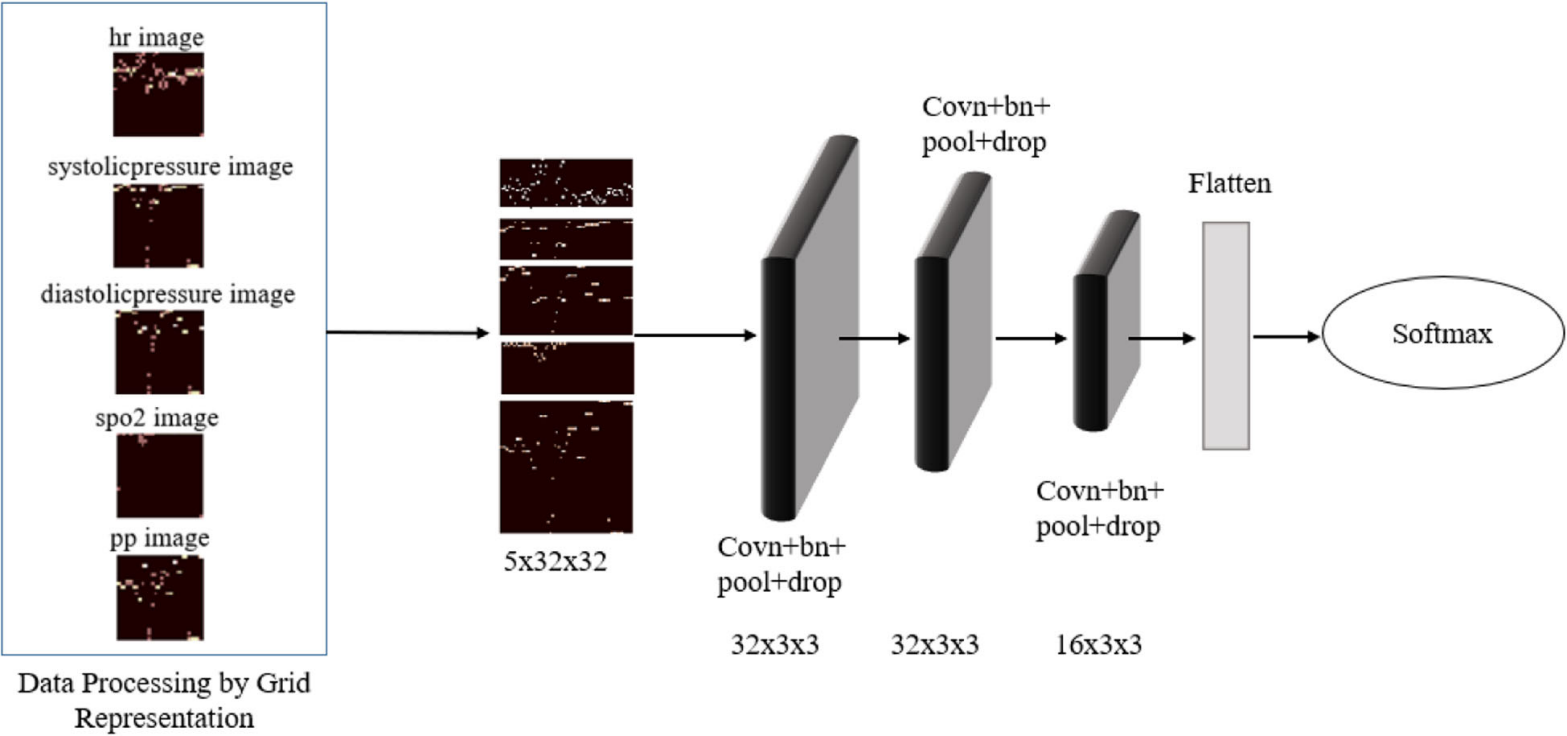
Prediction of heart failure risk based on image features

Although deep learning is now well developed in computer vision and speech recognition, it is difficult to build predictive models when it comes to time series. Reasons include that Recurrent neural networks are difficult to train and there are no existing trained networks for time series. But if we turn the time series into pictures and then we can take advantage of the current machine vision for time series. Therefore, we convert the vital sign data of the patient into grid image by using the grid representation, and then the convolutional neural network is directly used to identify the grid image for heart failure prediction in this paper. See Fig. 10.

The grid representation is a compression technique that we convert a time series to a matrix format. Given a time series X = { *x*_*t*_, t = 1, 2,..., T}, the length of which is T, and a grid structure, which is equally partitioned into m × n rectangles and the number of row and column are m and n, respectively, we are able to produce a grid representation as where *a*_*ij*_ is the number of data points located in the i-th row and the j-th column so it should be an integer and satisfies *a*_*ij*_ ≥ 0. See the algorithm for details [29]. A good representation method should retain as much information as possible of the initial time series when compressing it. Time series contain not only time and value information but also point distribution information. The m × n grid structure can meet these requirements, so a method of representing time series is introduced. In this paper, the values of m and n that we used for the similarity measure are dependent on the structure of CNN. We designed a small network structure because of the small dataset, and all samples used the same m and n.

The converted time-series grid image (see Fig. 4) is fused at the channel level as input to the convolutional neural network for heart failure prediction.

### Data description

**Fig. 11:**
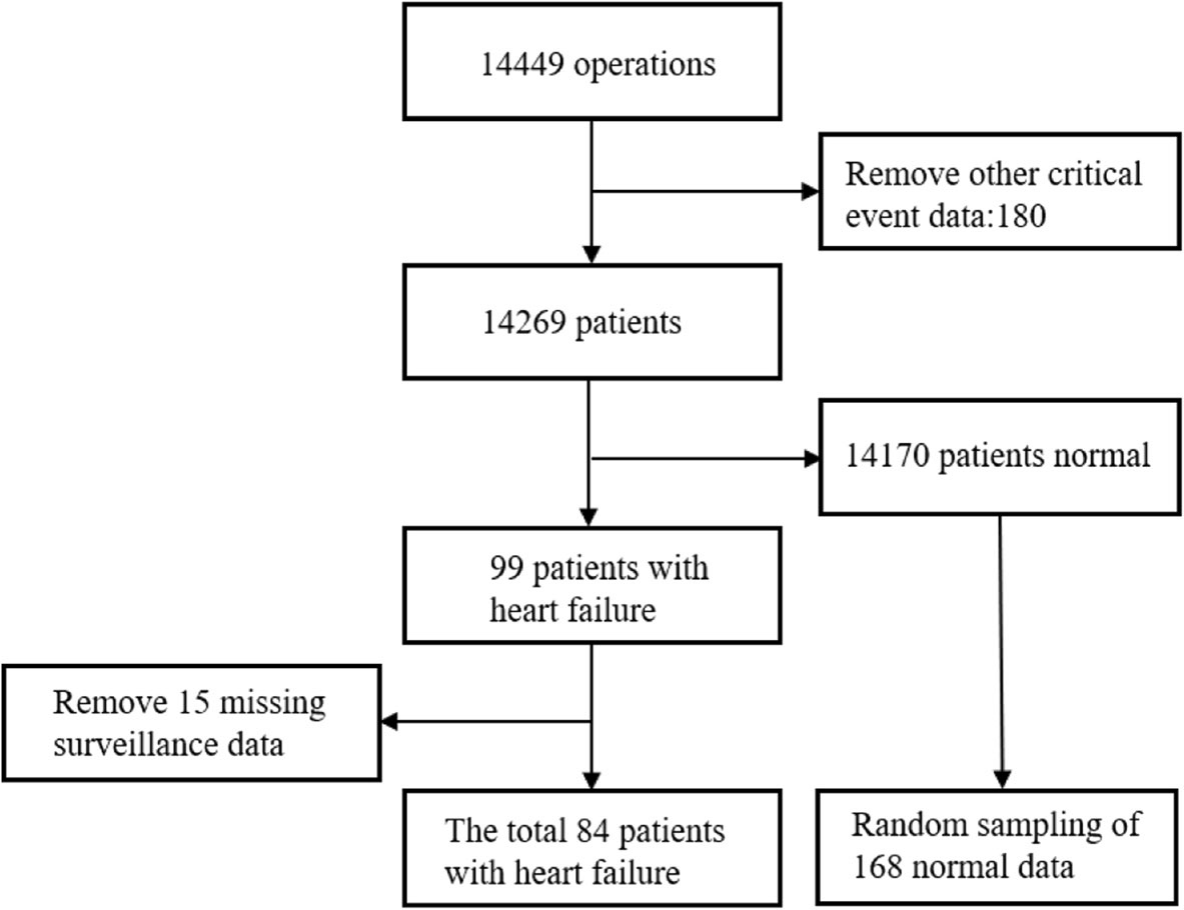
The data screening diagram

The data used in this paper is from the Department of Anesthesiology, Southwest Hospital. All data were gathered from the surgical patients from June 2018 to October 2018. A total of 14,449 operations include 99 cases of postoperative heart failure, 46 cases of liver failure, 61 cases of death, renal failure 54,49 cases of respiratory failure and 31 cases of sepsis. The remaining is uncomplicated patients. 15 out of 99 patients with heart failure had incomplete monitoring data. These patients were removed from the experiment and the remaining 84 patients were positive. 168 cases of negative data were randomly selected from the normal data set for the experiment. The training set is 80% and testing set is 20%, we used 10-fold cross validation in the experiment. Particularly, we divided the training set into training set (9 sets) and validation set (1 set), then used the test set to evaluate our model. The data screening diagram is as Fig. 11.

## Results

### Experiments based on statistical representation

**Fig. 12:**
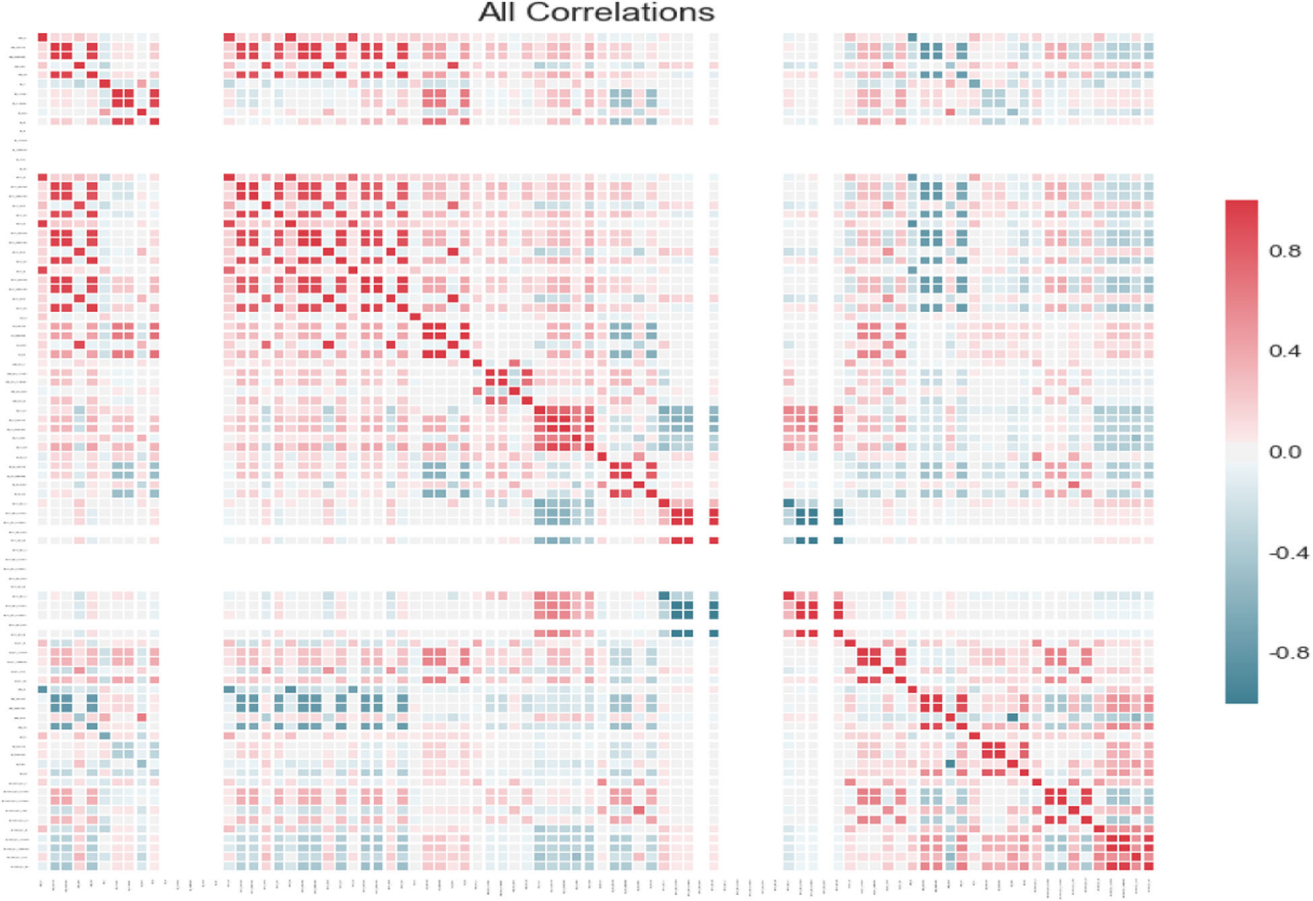
The correlation of each feature

The statistical features have a total of 90 variables, and the data has to be selected before prediction. In order to reduce calculation complexity, features with lower importance should be removed. In this paper, the correlation was analyzed that calculating the Pearson CorrelationCoefficient of each feature, then the features with importance of 0 were removed. Figure 12 shows the correlation of each feature, in which the regions with dark color tend to have a strong correlation and vice versa.

**Fig. 13:**
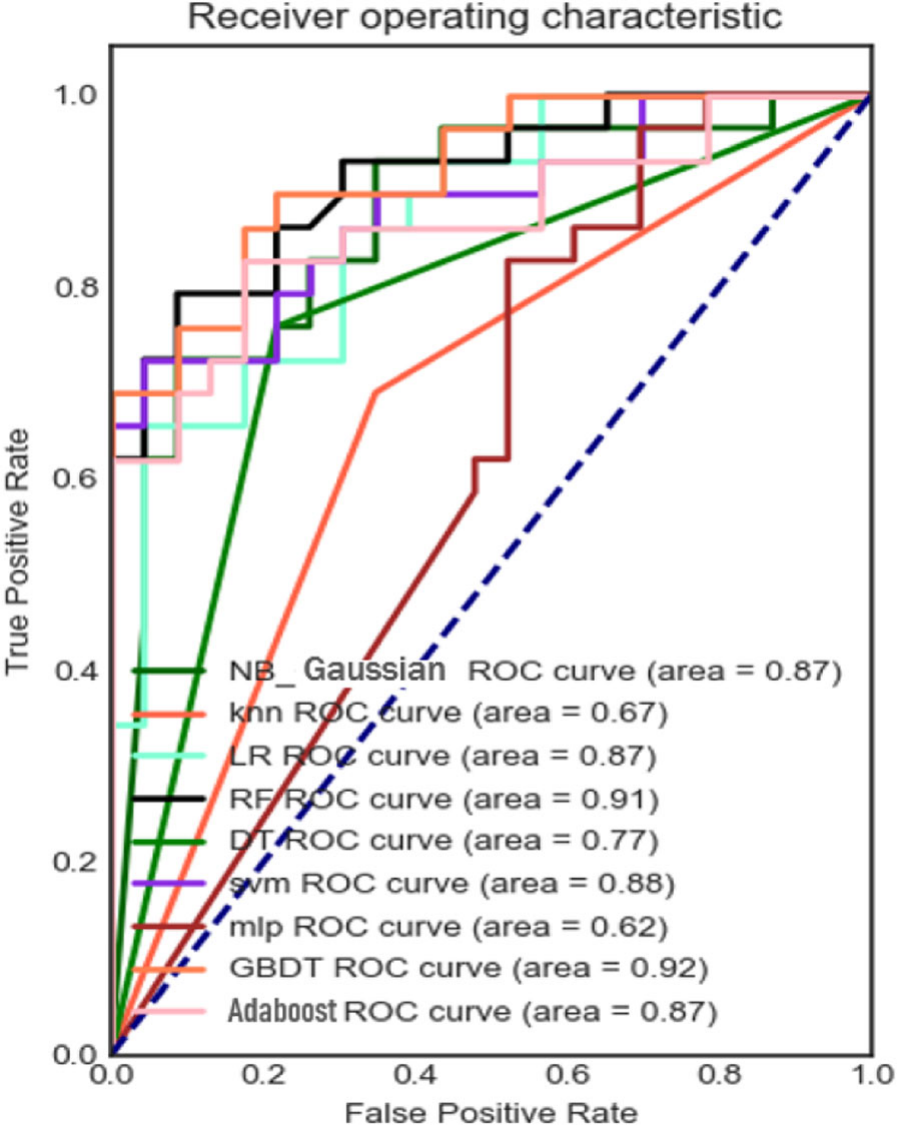
The ROC curve of 8 classifiers based on Statistical Representation

Models were built from these statistical features using 8 different classifiers: Adaboost, Decision Tree (DT), Support Vector Machine (SVM), Logistic regression (LR), naive Bayes (NB), Random forest (RF), Multiple perception machine (MLP), Gradient Boosting Decision Tree (GBDT). Because the sklearn library of python includes these machine learning methods, we used the sklearn library to build these models. The core principle of AdaBoost is to fit a sequence of weak learners (i.e., small decision trees) on repeatedly modified versions of the data. All the predictions are then combined by weighted majority voting (or summation) to produce the final prediction. The data modification for each so-called boosting iteration involves applying weights to each of the training sample. The parameter of Adaboost was: n_estimators is 100. Decision Tree is to create a model that predicts the value of a target variable by learning simple decision rules inferred from the data features, where “DecisionTreeClassifier” of scikit-learn is a class capable of performing multi-class classification on a dataset. The parameters of DT were: criterion is “gini”, min_samples_split is 2, min_samples_leaf is 1, min_weight_fraction_leaf is 0.0. SVM is a set of supervised learning methods used for classification, regression and outliers detection. SVM in scikit-learn supports both dense (“numpy.ndarray” and convertible to that by “numpy.asarray”) and sparse (any “scipy.sparse”) sample vectors as input. The parameter of SVM was: kernel is “rbf”. In the model of Logistic regression, the probabilities describing the possible outcomes of a single trial are modeled using a logistic function. Logistic regression is implemented in LogisticRegression. This implementation can fit binary, One-vs-Rest, or multinomial logistic regression with l2. Naive Bayes methods are a set of supervised learning algorithms based on Bayes theorem, whose “naive” assumption is the conditional independence between each pair of features of a given class variable value. Random forests achieve a reduced variance by combining diverse trees, sometimes at the cost of a slight increase in bias. In practice the variance reduction is often significant hence yielding an overall better model. In RF, each tree in the ensemble is built from a sample drawn with replacement (i.e., a bootstrap sample) from the training set. Furthermore, when splitting each node during the construction of a tree, the best split is found either from all input features or a random subset of size max_features. The parameter of RF was: n_estimators is 100. The MLP is a supervised learning algorithm that learns a function *f*(·) : *R*^*m*^ → *R*^*o*^ by training on a dataset, where m is the number of dimensions for input and o is the number of dimensions for output. Given a set of features X= *x*_1_, *x*_2_, *x*_1_, …*x*_*m*_ and a target y, it can learn a non-linear function approximator for either classification or regression. It is different from logistic regression, in that between the input and the output layer, there can be one or more non-linear layers, called hidden layers. The parameter of MLP was: hidden_layer_sizes is (5, 2). The GBDT is a generalization of boosting to arbitrary differentiable loss functions. GBDT is an accurate and effective off-the-shelf procedure that can be used for both regression and classification problems. The module “sklearn.ensemble” provides methods for both classification and regression via gradient boosted regression trees. The parameter of the GBDT was: n_estimators is 200. The other parameters of these models were the default parameters, see the Appendix for details. The results are shown in Table 2, and the Receiver Operating Characteristic (ROC) is shown in Fig. 13.

**Table 2:** Sensitivity (TPR), specificity (TNR), F1 score, accuracy (ACC) of various classifiers

Feature	Methods	TPR	TNR	F1score	ACC
Statistical Feature	Adaboost	0.83	0.83	0.83	0.83
	DT	0.76	0.78	0.77	0.77
	GBDT	**0.83**	**0.85**	**0.84**	**0.84**
	LR	0.76	0.7	0.73	0.73
	NB	0.72	0.78	0.75	0.76
	RF	0.69	0.96	0.81	0.81
	SVM	0.66	0.96	0.79	0.81
Text Feature	Adaboost	0.68	0.78	0.74	0.74
	DT	0.61	0.78	0.71	0.71
	GBDT	0.68	0.78	0.74	0.74
	LR	0.61	0.93	0.79	0.81
	NB	**0.84**	0.73	0.78	0.79
	RF	0.65	0.84	0.76	0.76
Image Feature	GRCNN	0.74	**0.89**	0.83	**0.83**

The entries in boldface indicate the best results for classifiers in three learning methods. Specifically, these results demonstrate the GBDT classifier achieves the best results in the prediction of heart failure by statistical feature representation. The sensitivity, specificity and accuracy are 83, 85, 84% respectively; the NB classifier achieves the best results in the prediction of heart failure by text feature representation. The sensitivity, specificity and accuracy are 84, 73, 79% respectively; The sensitivity, specificity and accuracy of classification prediction based on convolutional neural network in image feature representation also reaches 89, 78 and 89%, respectively

### Experiments based on text representation

**Fig. 14:**
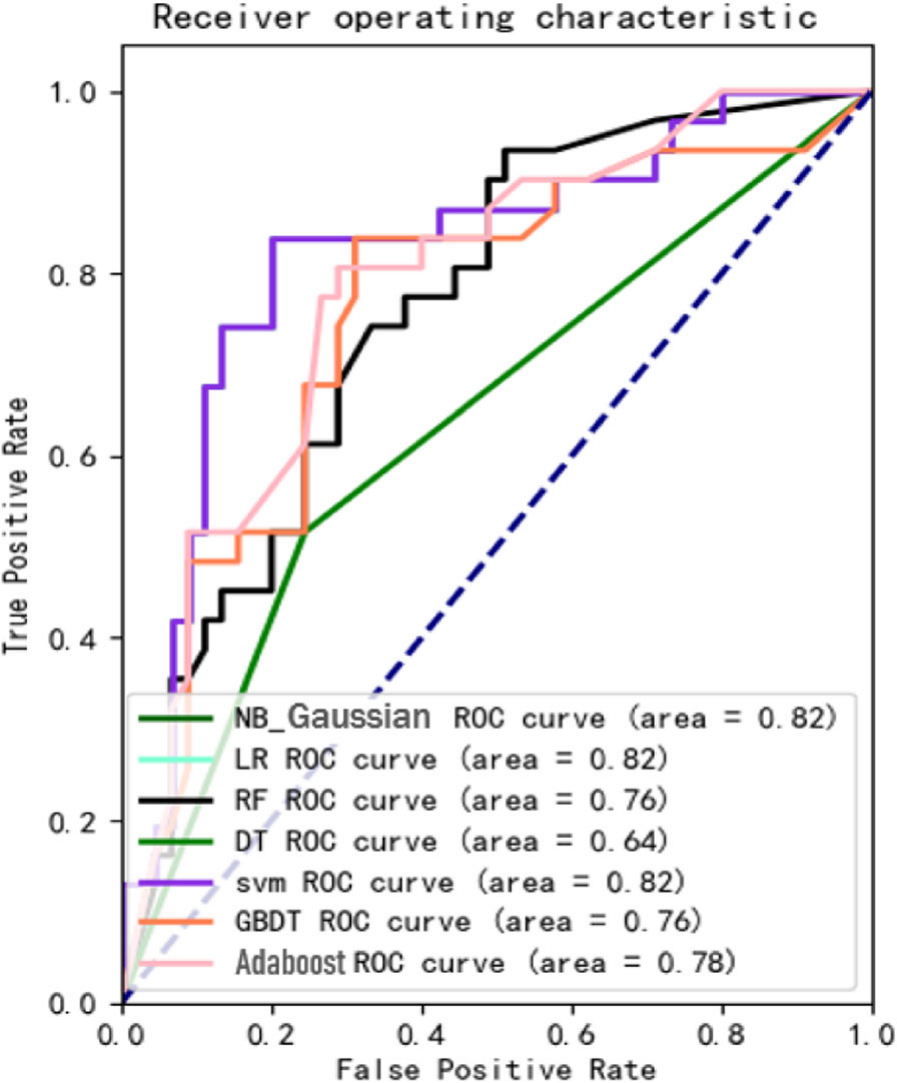
The ROC curve of 8 classifiers based on Text Representation

Figure 9 provides a general overview of our experimental process. First, we convert the patient’s vital signs monitoring data for 3 min into alphabetic symbols and convert consecutive 3 alphabetic symbols to text based on the rule engine. The LDA was used to unsupervised cluster all patient’s text representation into 5 topics. We chose 5 topics after varying the number from 2 to 10, because it was noted that validation set accuracy did not improve after 5, so that each patient’s vital signs monitoring data is represented by a 5-dimensional vector, summing to 1. Finally, we performed heart failure prediction based on the representation of the topic probability distribution using the same classifier and parameters as the Statistical Representation. The experimental results are shown in Table 2, and the ROC curve of the experiment is shown in Fig. 14.

### Experiments based on image representation

In this experiment, we first convert the patient’s heart rate, diastolic blood pressure, systolic blood pressure, spo2, and pulse pressure difference into the grid image, and fuse the five images in the channel layer as input to the convolutional neural network (see the network structure designed in the previous section. See Fig. 11) to extract image features. Finally, heart failure is classified by softmax.

6 $$ \left(5,\mathrm{L},1\right)=>\left(5,\mathrm{m},\mathrm{n}\right) $$

**Fig. 15:**
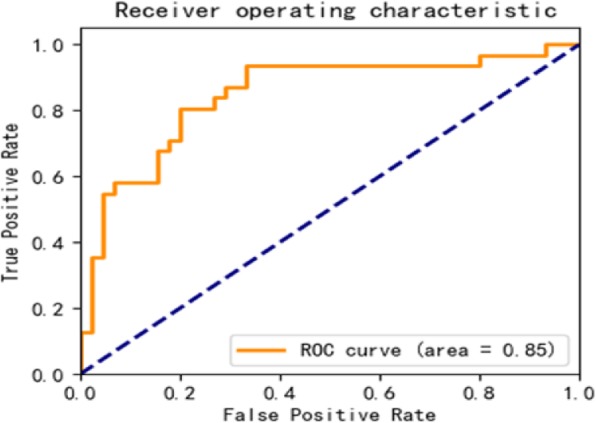
The ROC curve of CNN based on image representation

**Fig. 16:**
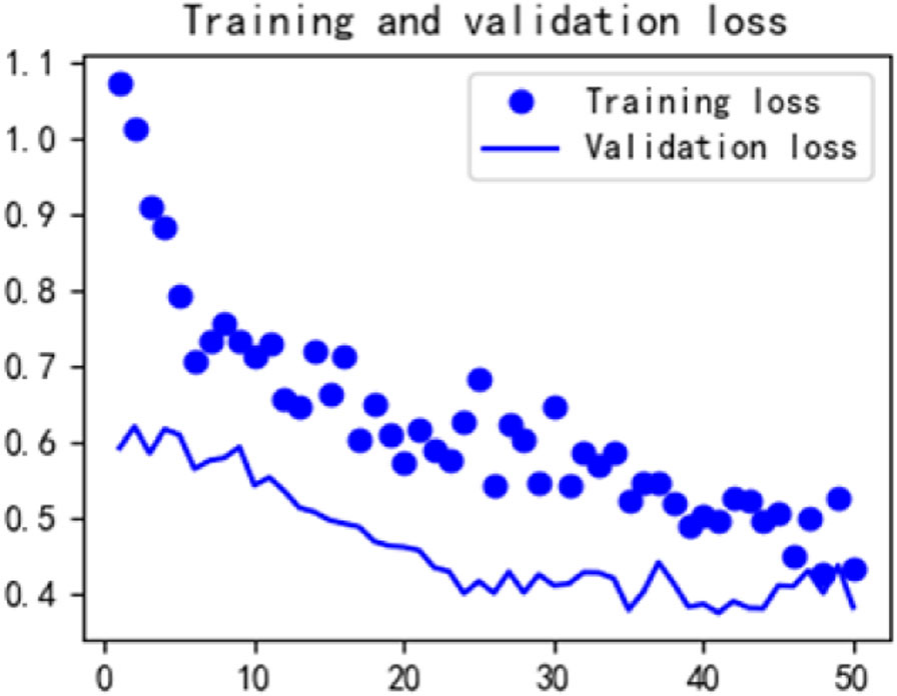
The loss of training and validation of convolutional neural networks

**Fig. 17:**
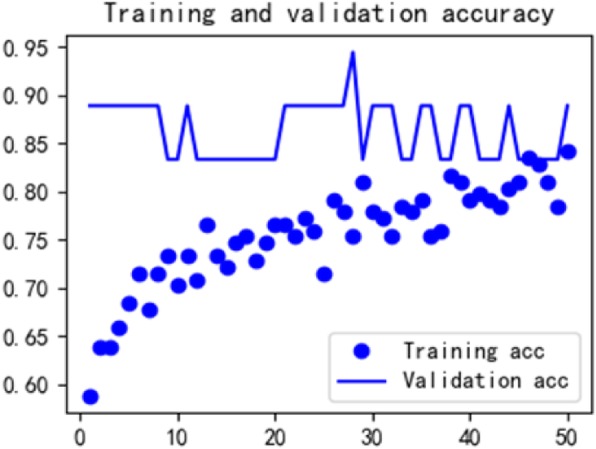
The accuracy of training and validation of convolutional neural networks

See Formula 6, where L is the length of the monitoring time series data, and (m, n) is the width and length of the grid image. The converted image has an associated length and width. Five grid maps of each patient simultaneously input into a convolutional neural network for heart failure recognition. The experimental results are shown in Table 2, and the ROC curve of the experiment is shown in Fig. 15. Figures 16 and 17 show the loss and accuracy of training and validation of convolutional neural networks.

Predictive results of various feature representations are presented in Table 2. These results demonstrate the GBDT classifier achieves the best results in the prediction of heart failure by statistical feature representation. The sensitivity, specificity and accuracy are 83, 85, 84% respectively; the NB classifier achieves the best results in the prediction of heart failure by text feature representation. The sensitivity, specificity and accuracy are 84, 73, 79% respectively; The sensitivity, specificity and accuracy of classification prediction based on convolutional neural network in image feature representation experiments also reached 89, 78 and 89%, respectively. It can be seen from Figs. 14, 15 and 16 that the AUC values based on the three feature representation algorithms are 0.92, 0.82, 083 respectively. Therefore, from the overall results, the patient’s intraoperative vital signs monitoring data has the ability to capture the precursory information of heart failure during the perioperative period.

Among the three feature representations, the method based on statistical representations achieves the best results. Because we did a lot of feature engineering before the model prediction, we removed the low-importance features and only retained the relevant features. In addition, the total sample size of the experiment is only 252 cases (positive: 84, negative: 168). Small sample size based on traditional feature engineering can achieve better results in classification. However, the method of text and image feature representation based on LDA and convolution neural network is likely to have the problem of under-fitting in the small sample training data set. Therefore, there should be a lot of room to improve the experimental results.

## Discussion

Heart failure in the perioperative period is one of the most significant causes of postoperative death of patients. At present, because the valuable diagnostic indices of heart failure have lagged effect, which are often used only for differential diagnosis after adverse events have occurred, and are difficult to be used for early diagnosis and prediction, the early clinical diagnosis of adverse events of heart failure still relies on the clinical experience of anesthesiologists and physicians. Therefore, there is a lack of early intraoperative prediction techniques for perioperative adverse cardiac events. Previous studies have shown that the direct monitoring data in operation has the value of early diagnosis and early warning after preprocessing and analysis of time series data. However, as far as we know that there is no direct use of intraoperative monitoring signs data on patients with perioperative risk prediction of heart failure. Thus, our method is the first study to predict perioperative heart failure using only intraoperative monitoring of vital signs.

At present, much literature in heart failure prediction and diagnosis has focused on using ECG data and bio-marker as input to a classifier. Because the heart failure prediction is more difficult than diagnosis, the methods of heart failure diagnosis usually achieved a better performance, such as: AUC of 0.883 (Choi et al. [7]), the classification accuracy of 96.61% (Chen et al. [11]). However, the methods of heart failure prediction usually achieved a poor performance, such as: the sensitivity of 0.42 (Petersen et al. [14]), the predicted AUC reached 0.82 (Koulaouzidis [8]), the predicted AUC of 0.78 (Shameer et al. [9]), the prediction accuracy of 78.4% (Zheng et al. [10]). Our work differs in that we only consider intraoperative monitoring of vital signs to predict the risk of heart failure, and the sensitivity, specificity and accuracy of the best method can reach 83, 85 and 84% respectively. It demonstrates that using only intraoperative monitoring of vital signs data can largely predict the risk of heart failure, and reach high accuracy. It shows a valuable potential to save the life for heart failure patients using intraoperative monitoring of vital signs.

There are several limitations of this body of work. Firstly, prediction method based on text and image features is ineffective because of too few experimental samples. The model proposed in this paper can’t clearly determine the specific correlation between intraoperative vital signs monitoring data and heart failure. Future directions for this work should include new model to clarify the correlation between the two and we could also improve the prediction quality of our model with additional features, such as relevant preoperative examination indicators, etc. In the future, we hope that such methods will be used to provide medical staff with the support to improve decision making for surgical surgeon.

## Conclusion

In this work, we proposed three machine learning methods including statistical learning representation, text learning representation and image learning representation to process vital signs monitoring data (heart rate, systolic pressure, diastolic pressure, blood oxygen saturation and pulse pressure) for estimating the risk of heart failure. The method was evaluated by monitoring data of perioperative patients in anesthesiology Department of Southwest Hospital. The results of our experiment demonstrated that the representation learning model of vital signs monitoring data in intraoperative patients can capture the physiological characteristics of heart failure in the perioperative period. Additionally, these results showed that the GBDT classifier has achieved the best results in predicting heart failure by statistical characteristics. The sensitivity, specificity and accuracy of the best method can reach 83, 85 and 84% respectively. Therefore, we can draw a conclusion that the patient’s intraoperative vital signs monitoring data has the ability to capture the precursor information of heart failure in the perioperative period, which is important for reducing the risk of heart failure and improving the safety of the patient. Furthermore, this paper shows a valuable potential to develop modern medical diagnosis and treatment by using vital signs monitoring data in intraoperative patients for risk prediction of the perioperative adverse cardiac events.

## Data Availability

The raw data required to reproduce these findings cannot be shared at this time as the data also forms part of an ongoing study.

## Appendix

**Table 3:** The model parameters

Model	The parameter settings
Adaboost	n_estimators = 100 # The maximum number of estimators at which boosting is # terminated.
Decision Tree (DT)	criterion = “gini” # The function to measure the quality of a split, supported criteria # are “gini” for the Gini impurity min_samples_split = 2 # The minimum number of samples required to split an # internal node. min_samples_leaf = 1 # The minimum number of samples required to be at a leaf # node. min_weight_fraction_leaf = 0.0 # The minimum weighted fraction of the sum total # of weights required to be at a leaf node.
support vector machine (SVM)	kernel = “rbf” # Specifies the kernel type to be used in the algorithm. # “rbf” is Gaussian kernel function.
logistic regression (LR)	penalty = “l2” # Specifies the norm used in the penalization, the ‘l2’ penalty is the # standard used in SVC.
Random forest (RF)	n_estimators = 100 # The number of trees in the forest.
Multiple perception machine (MLP)	alpha = 1e-5 # It is regularized parameters. hidden_layer_sizes = (5, 2) # The i-th element represents the number of neurons # in the i-th hidden layer.
Gradient Boosting Decison Tree (GBDT)	n_estimators = 200 # The number of boosting stages to perform.

## Abbreviations

ACC: Accuracy
AUC: Area under the curve
CNN: Convolutional Neural Networks
Conv: Convolution
diff: Difference
DL: Deep learning
DT: Decision Tree
ECG: Electrocardiograph
GBDT: Gradient Boosting Decision Tree
GRTS: Grid Representation for Time Series
HR/hr.: Heart rate
kurt: kurtosis
LDA: Latent Dirichlet Allocation
LR: Logistic regression
max: Maximum
min: Minimum
MLP: Multiple perception machine
NB: Naive Bayes
NIDIASBP/nidiasbpe: Diastolic blood pressure
NISYSBP/nisysbp: Systolic blood pressure
NYHA: New York Heart Association
PAA: Piecewise Approximate Aggregation
PAA: Piecewise Approximate Aggregation
perc25: 25%
perc50: 50%
perc75: 75%
Pool: Pooling
PP/pp.: Pulse pressure difference
RF: Random forest
ROC: Receiver Operating Characteristic curve
SAX: Symbolic Aggregate Approximation
SAX: Symbolic Aggregate Approximation
skew: Skewness
std.: Variance
SVM: Support vector machine
TNR: Specificity
TPR: Sensitivity
TSC: Time Series Classification
